# Mental Dynamics, in Relation to the Science of Medicine

**Published:** 1852-07-01

**Authors:** Stanhope Templeman Speer

**Affiliations:** PROFESSOR OF PHYSIOLOGY IN THE UNIVERSITY OF MONTPELLIER; CHELTENHAM


### MENTAL DYNAMICS, IN RELATION TO THE SCIENCE OF MEDICINE.

A COURSE OF LECTURES DELIVERED BY M. LORDAT, PROFESSOR OF PHYSIOLOGY IN THE UNIVERSITY OF MONTPELLIER. ARRANGED AND TRANSLATED BY STANHOPE TEMPLEMAN SPEER, M.D., CHELTENHAM.

### Lecture I.

A remarkable contrast exists between the rival schools of Paris and Montpellier, in their mutual appreciation and explanation of what I denominate the "principle of intelligence." It is but a few years, since the heads of the profession in the capital of France knew nought of the nature of man but what was to be found in bodies submitted to inspection after death. Convinced that our existence was but a matter of tissues and systems, they persisted in investigating the science of medicine by a close attention to the condition of the corporeal organs.

Such a belief, however, has been but of comparatively short duration. Their want of success in the above mode of prosecuting so important a research; their inability to reconcile certain well-founded precepts in therapeutics, with anatomical rules; the variability of their doctrines contrasted with that of a school which advances, slowly indeed, but surely; all these, I repeat, have at length led them to the conclusion, that they have chosen a wrong path.

The press of the French capital affords us a striking example of the difficulty then experienced in founding an anthropology capable of forming a sound basis for the science of medicine. The materialists readily perceive the difficulty of solving the intricate problem, of which man, in his fullest sense, is a personification. They admit that anatomy, physics, chemistry, and mathematics, fail to give an idea of the human dynamism.

It is something to have allowed that man is not formed solely of materials appreciable by chemistry and physics. The conviction of an error is the first step towards its repudiation. But more than this is requisite, to elevate the art of medicine to the dignity of a science. The compass hitherto trusted to, has been cast aside, without another being at hand to steer by. It would appear, indeed, that the school of which I have just spoken, does not possess any scientific method capable of being successfully opposed to that, for instance, which is familiar to the pupils of this university.

In a discourse lately emanating from the press of the capital, the orator, after acknowledging the insufficiency of physical science in explaining the phenomena which occur in the living body, takes refuge in uncertainty, rather than have recourse to any other method of investigation. He thus expresses himself:— "To the chemical theories of the present day, which outrun the actual discoveries made by the science itself, must we oppose the abstractions of vitalism, which merely forges a physiology independent of organs, and even then accords it but an accessory importance?"

Now it appears to me, that this sentiment may be taken as the expression of scientific opinion among the medical observers of this country; and it indicates, first, a complete forgetfulness of the philosophy of Bacon, of so much importance in its application to medicine; and secondly, an ignorance of the real and essential nature of vitalism, one of whose chief characteristics is to examine, with the same zeal and the same unbiassed judgment, causes, some of which are appreciable by the senses while others are invisible; so that, with us, the whole of man's nature is equally deserving of inquiry—we see in it nothing accessory.

Now this forgetfulness of the rules of philosophy, as laid down by Bacon, or the inability to carry them out in practice, would appear to be the principal cause of this uncertain stagnation in medical opinion. Materialism, once in such favour, has been now abjured as an absurdity. Ashamed of being made the dupes of physical theories, relative to the principles of life and intelligence, its votaries no longer repose confidence in it.

To remedy this, the simplest plan would appear to be a return to the doctrines of Hippocrates, so rashly cast aside about the middle of the seventeenth century.

On a former occasion, I spoke of the disorder occurring in the world of medical opinion, at the time of the Cartesian revolution; and you must be well aware, that in all cases of commotion, order is seldom re-established without some injury having taken place. It is indeed difficult for the mind, freed from its previous shackles, to avoid abusing its newly-found liberty. In cursing the prejudices by which it had been hampered, how many truths may it not entangle in the proscribed objects of its hatred. Hippocrates had laid it down that man was a combination of three causes; the first, an aggregate material; the second, a principle of intelligence, incommensurable with such an aggregate; the third, a vital force, which is neither the one nor the other, and is to be appreciated solely by its effects. Now this distinction, so long upheld, was at length abolished upon the authority of Descartes, and ere long, the science of human metaphysics experienced the ill effects of his teachings; it first underwent mutilation; was then attacked, scouted, and ridiculed; and of medical science there was soon left but anatomy and surgery.

We have seen, however, that this state of things did not, and could not last, and that a return to the science of metaphysics became inevitably necessary. Why, however, was this science, as taught by Hippocrates, so studiously shunned? I may be allowed to explain it in the following manner.

The Hippocratic doctrine, so admirably inculcated and practised in Paris, by Bailliou, at the time of the Cartesian secession, was rather too rudimentary, and at the present day, more especially, bears too strongly a gothic origin to be at all presentable. There exists, however, a modification of it, that has gradually expanded, cotemporaneously with the advance of civilization, has taken proper cognizance of every discovery, and has armed itself with all the varied weapons that have ever and anon been directed to its annihilation; but its most enthusiastic champions happen to belong to that school which the admirers of Cabanis, Bichat, and Broussais have long spurned and insulted, and which they now regard with sullen dislike.

Be this, however, as it may, let us take some examples to prove that many of the most inveterate materialists have at length recognised and acknowledged the reality of causes of a metaphysical nature, the duality of which, however, they still deny.

At a meeting of the Academy of Sciences, one of the physicians of the Salpêtrière, M. Lelut, allowed the inability of morbid anatomy to throw light upon the theory of mental affections, and openly confessed that the various diseases coming more especially under his own observation, and the theory of which it was a duty to solve, were under the influence of certain invisible causes, totally irrespective of organs; he likewise declared that a necessity existed for seeking some metaphysical explanation of such phenomena. After reading his oration, however, it is easy to see that the author is a spiritualist of the Cartesian school. The principle of intelligence is, in his eyes, a speciality incompatible with matter; and as regards that vital force which, while unconscious of itself, possesses, nevertheless, a unity and activity of its own, and which presides over the development of the body, and preserves it from a thousand disturbing influences—with regard to this force, I repeat, he entertains a hope that it may yet be explained by a reference to physical laws, whenever anatomy and microscopy shall have taught us the secret of the union of the different nervous fibres. All that Stahl has ever urged against the possibility of such a disclosure, would appear, with M. Lelut, to be as nought.

Again, M. Collineau, in his "*Analyse Physiologique de l'Entendement Humain,*" raises his voice not only against psychological materialism, but against vital mechanism likewise. Unfortunately he appears to have misunderstood the duality of the human dynamism, and to have adopted that bastard description of Stahlianism, still adhered to by a few, who, while acknowledging the absurdity of materialism and the necessity of a cause of metaphysical origin, will not allow, with Stahl, that life is the expression of a spiritual entity, which deteriorates as we descend in the scale of the organic world, from man down to the lowest extremity of this domain; but assert that the principle of life is the radical base and essence of every living thing, and that human intelligence is but a high degree of this very principle. Upon this hypothesis is the work of Collineau based, and it is but a reversed form of animism, applicable in case of necessity to organicism on the one hand, and to Hylosism, or materialism proper, on the other.

I might adduce additional examples to show that many of our Parisian brethren have renounced the doctrines of materialism, and have recognised the existence of a human dynamism, based on metaphysical doctrines; unfortunately, this laudable confession has led to no results, having become a mere hypothesis, simply from an obstinate determination to include under one and the same cause, two series of phenomena which common sense alone would suffice to separate. I mean by this, that the distinction between the two actually existing order of causes is regarded in the light of an hypothesis or opinion.

But the duality of the human dynamism is not an opinion; it is a fact deduced from daily experience. Inductive philosophy forbids us to say that the vital force and the principle of intelligence are of one and the same nature. Of the importance of this truth you will, I am sure, be persuaded, when you learn that it is the base of the genuine Hippocratic medicine.

In proportion, then, as this doctrine is neglected, do I consider it imperative to bring it before the mind of my hearers, and I purpose devoting a portion of the present course to its elucidation.

The term life, as understood by naturalists, is a temporary phenomenon, consisting in the formation, growth, degradation, and dissolution of a certain mixed aggregate. In this we must distinguish the material element which falls under the cognizance of our senses, and a mysterious dynamism, constituting the active agent in the production of vital phenomena.

This aggregate and its vivifying influence commence by a point of origin scarcely appreciable. They increase progressively up to a certain epoch, say forty years; the acting force then decreases, by a gradation similar to that which marked its previous augmentation; and the aggregate material undergoes a simultaneous degradation.

After a certain period of time, this same vital force finds itself on a level with its primary origin, and shortly afterwards becomes extinct. The material aggregate has not been destroyed, but from the first moment of its deterioration it has imbibed a tendency to decay. Endeavours may have been made to repair the ravages of time, but such conservative efforts have served but to ward off the inevitable termination. The tenement was still habitable when the vital force became extinct; and the material aggregate, no longer under any directing vivifying influence, was seized upon, disintegrated by the action of the elements, and its component parts dispersed. Thus, in a zoologic point of view, the life of man, like that of many animals, presents two qualitative phenomena—a progressive increase, which we may designate youth, and a progressive decrease, denominated old age. The period which marks the cessation of increase or of youth, and the commencement of decrease or old age, is well deserving of a special denomination, which I shall borrow from one of the technicalities of astronomy. If, then, the creative power of life ascends, as it were, to a certain point, its meridian, it must culminate previous to descending; and when I shall speak of the vital force being actually at the culminating point, you will understand that I allude to that period at which, having attained its maximum of perfection, it is about to return to its pristine condition.

If, now, we turn from this view of the question, and look closely into the object of our present investigation, man; we perceive that this vital force, which is the author and promoter of all vital phenomena, and is at the same time unconscious of its own existence, undergoes a progressive increase, a culminating period, and a finally gradual declension . . . . but that the principle of intelligence, self-existing, self-conscious, self-appreciable, and capable of ignoring the operations of the vital force . . . . alike experiences a gradual increase, but not the culmination of this latter force, since it is not liable to a process of decay, but continues its onward progressive course.

The contrast thus manifested at so important a period of our existence, does it not indeed merit the most profound attention? If senescence be the appendage of a vital principle, and that, on the other hand, the intellectual principle be irrevocably exempt from it—such an immunity, would it not suffice to prove that the former of these processes was essentially of a nature different from that of its coadjutor?

Such, however, has not been the opinion of many ancient and modern writers; among the former we find Lucretius. . . . After premising that the principle of intelligence is an aggregate material, of a nature identical with that of the body in which it resides, he thus continues: "We see it originate simultaneously with the body, increase and grow old along with it. During infancy, a frail and feeble organism serves as the cradle for an equally feeble intelligence; age, in strengthening the limbs, ripens the intellect and increases the vigour of the mind. Lastly, when the influence of years has altered the form of the body, blunted its perceptions, and exhausted its powers, the judgment wavers, and the mind partakes of the uncertainty of the speech. In a word, all the springs of the machine give way in turn. Is it not, therefore, reasonable that the mind should undergo a like prostration, and end its career as smoke borne upon the wings of the wind, since we thus see it arise, increase, and finally succumb, beneath the pressure of accumulating years?" (Lucretius, *De Rerum Natura*, lib. iii.)

Another poet, Shakspeare, has reproduced the same idea in a manner by no means worthy of his great reputation. The description which he gives of the seventh age, and of its alleged characteristic, imbecility, I must take leave to say is false. Imbecility is not the result alone of old age, since it may supervene at any period of our existence; and even were it true that the decline of life is especially liable to it—a point upon which statistics are not as yet agreed —it is certainly not the fact, that one characteristic of old age is a reduction of the intellectual principle to the standard of the newly-born infant.

A philosopher of our own period, Jouffroy, in his "*Mélanges Philosophiques*," has likewise adopted a similar view of the decline of the intellectual principle, and its return to the infantile condition. "When," says he, "man attains a great age, he usually finishes where he began; that is, in the impersonal vitality which precedes during infancy the birth of the will, and hence the ordinary expression, that the old man has become a child—has entered his second childhood."

This assertion is then developed with considerable talent, without, however, any proof being adduced; of which, however, the author does not perceive the necessity—since the fact is, according to his opinion, a matter of observation, or, in other words, a popular persuasion.

It is against this persuasion that I raise my voice, and upon which I demand some more ample information. The formal intention of Epicurus and his followers, has been to disseminate a belief that the principle of intelligence is identical in nature with the body it inhabits; that the mind and the material aggregate, or body, are co-partners; that their operations are subject to the same vicissitudes, and that the development, the growth, the deterioration of the different parts of this aggregate, are accurately imitated in the operations of the intellect. But an impartial method of daily observance will, I conceive, suffice to falsify and disprove that doctrine.

For this purpose, let us examine a certain number of individuals well stricken in years, and whose intellect has been thoroughly cultivated; let us listen to their avowals relative to their intellectual condition, and let us, at the same time, examine their actions. Let us not choose, however, one who, like Le Sage's Bishop of Grenada, after a succession of apoplectic fits, ended by deceiving himself relative to the consecutive infirmities he had experienced as their result. But we may take as an example a healthy sexagenarian, who after having thoroughly tested his intellectual and vital powers, honestly confesses what are actually the respective conditions of these two principles. To appreciate satisfactorily this species of observation, it should be conducted in such a manner as to enable us to verify the resulting declaration by the subsequent acts of the individual in question. The subject for such an observation has long been at hand, it remains but to examine it carefully. I allude to Bossuet. He possesses all the required desiderata, and we take his declaration, made during his sixtieth year; up to that period his life had been one, marked by laborious exertions of every description, and of his sincerity there can be but one opinion. The eighteen years of his life which succeed the epoch of which I speak, belong to the history of the human intellect.

The declaration to which I now refer is so well known, that there is perhaps not one of my auditors to whom it is not familiar. It forms the close of his oration over the body of the great Condé. It will be for us an easy matter to abstract, intellectually, all that pertains to the religious faith of the orator, and to confine our attention solely to the avowal he makes relative to his past, his present, and his future; you will remember that his peroration is a prosopopœia addressed to the departed hero, and he thus expresses himself:—"You here assign a termination to these my funeral orations. Instead of deploring the death of others, I will learn of you, O mighty prince, to make my own holy. Happy, if warned by these grey hairs, of the account I must soon render of my stewardship, I may reserve for the flock, to whom it is my duty and mission to dispense the words of eternal life, the relics of a voice already quavering, of an ardour almost extinct."

This exordium, the effect of which upon the hearers of Bossuet is still to be seen in the admiration of his readers at the present day, we shall now endeavour to reduce to a simple expression of facts, divested of the flowers of oratory. Let us imagine for an instant, Bossuet communicating the same ideas in conversation with an intimate friend, without style, solemnity, or allegory, but in language dry and prosaic as that of the natural historian; candid as from brother to brother. Let us, then, attempt to paraphrase this group of ideas.

"My last discourse has now been pronounced. The success that has attended my efforts in the midst of the most illustrious of the world's assemblies, has depended as much on delivery as upon the sentiments uttered. But I have now lost the major part of those physical and vital qualities necessary for the production of such effects. Years have mutilated my corporeal qualifications, wrinkles have destroyed the play of my features, my eyes have lost their wonted fire, my voice and speech have become uncertain and quavering. I want words to express my ideas; and yet these ideas exist in all their perfection. Nay, since the period of my corporeal degradation, the mind has seemed to grow by a daily acquisition of new ideas. The more this frail body invites seclusion and care, the more does the principle of intelligence urge me forward to action. Happily, I adore the profession which I have so long cultivated, and I believe that my mission (which is that of diffusing its precepts) may still prove of service to the cause of morality. But I shall no more appear on the scene of so many former triumphs, upon which I can no longer expect to elicit either pleasure or emotion. I shall continue, nevertheless, in a career which permits me still to instruct the world by improving science, and my parishioners, whom I love, by a communication of ideas delivered in a simple amicable style. In this way only may I hope to negative the growing inutility of the body, and to prove a source of benefit, as long as it remains above the sod."

This amplification, then, is it not, I ask, a true exposition of the sentiments and intentions of this great man? Not only does it interpret all the fundamental ideas contained in his discourse, but it recalls all that the actual expressions appeared to predict. Bossuet fulfilled indeed his promise. The great orator no longer appeared on the stage of the world, but in the exercise of his pastoral functions, and through the medium of the press. Though he ceased to address himself to the admiring ears and eyes of the multitude, he directed his remaining energies to the care of their immortal souls. Neither Paris nor the French court ever more heard his thrilling accents, but eighteen years of his subsequent existence were occupied in disseminating through the religious world a prodigious number of writings, books, memoirs, dissertations, exhortations, and warnings, the merit of which appears to have progressively increased; and the entire collection of which is now honourably deposited as an item in the principal trophy of our country's glory.

It was during this second period of his existence, that Bossuet was placed among the foremost rank of orators, historians, dialecticians, philosophers, and fathers of the church.

Choose we any topic of a serious nature upon which to fix our attention, and we find in his writings the finest model of a thinking mind, and of the mode of arranging and transmitting our ideas. What, then, can be the meaning of those philosophers, poets, and physiologists, who assert that the principle of intelligence undergoes in old age a deterioration similar to that of the vital principle? The example I have just quoted, does it not suffice to repudiate such an assertion. Yes! In Bossuet, the unconscious vital principle, after attaining a culminating point, experienced its natural and expected degradation. Troubles, labour, and suffering may have, indeed, accelerated its extinction, and may even, if you will, have reduced it to the condition of an embryo. But as regards the principle of intelligence, it had lost none of its perfection, none of its utility, when death claimed him. On the day on which he ceased to breathe, his intellect was as powerful as when, forty years previous, Madame de Sévigné thus wrote respecting him, "Bossuet would appear as if engaged in a war of words with his auditors; his sermons are all mental combats."

You will perceive from what I have just said, that it has been, and ever will be, my intention to oppose that popular prejudice, which pretends that the principle of intelligence is amenable to the laws of age as is the vital force. This I undertake to refute, and my chief argument will rest upon a consideration of actual facts. The one I have already laid before you carries great weight along with it. But I shall not content myself with a single observation, which our adversaries might consider as exceptional. Not but what I might reasonably oppose it, and it alone, to any theory connected solely with causes of a physical nature, and we have already seen that our antagonists admit none other; for to prove that the decline of the principle of intelligence in man, is the result of the same cause to which we attribute the supervention of corporeal decay, it would be necessary to show that its declension was general, infallible, and free from exception. One single instance of immunity well authenticated, one well recorded case, in which the principle of intelligence remained unscathed during the senescence of its corporeal tenement, would suffice to destroy a theory, founded solely upon mechanical hypothesis. But as the public prefer reckoning the number of examples, rather than judging of each by its individual value, I am enabled to gratify the tendency, and to multiply facts to the utmost.

In undertaking the refutation of a theory, which the obstinacy of a sect may possibly protract, I fear lest the idea should suggest itself—where is the advan-

Insenescence = the power of not becoming old but he is silent to its insenescibility tage, where the utility, of proving that the principle of human intelligence does not suffer the inevitable decay of the vital force? Is it not a speculative question? Does the solution of the problem in any way tend to a practical application?

I have already afforded you a glimpse of the importance and extent of the proposition which I now advocate. But I fear lest this rapid sketch should not suffice to fix your attention as continuously as I should wish, and I have therefore chosen a certain number of examples, bearing upon the truth of this subject, and more especially capable of illustrating the applications of which it is most susceptible. As in the mutual interchange of thought a community of interest on either side facilitates the process, I shall not conceive it a loss of time, if I take some pains to place you in the most favourable light for seeing with ease, that the insenescence of the principle of intelligence becomes an established fact, an experimental dogma, capable of affording an insight into some important points connected with that science upon which medicine is based. Allow me, then, to point out to you a few applications of my proposition, in order to banish from your minds all doubts as to its utility.

My primary object, then, is to show that the principle of human intelligence does not experience that process of culmination to which the vital powers are inevitably subjected. I do not, however, seek to prove that this principle is in its own nature, by its own essence, incapable of growing old, for of this my own experience affords me no proof, and it is my especial wish to teach you nothing of which such experience has not satisfied me of the truth. A captious hearer might indeed say,—"I allow that you may meet with a number of individuals whose intellect has braved the ravages of time, but this does not prove anything beyond the simple fact, that the culminating point of which it may be susceptible is more tardy in its approach, and that the natural term of life has drawn to a close even before the principle of intelligence had attained its meridian." Gentlemen, to this I could say nothing, and as regards the insenescibility of this principle I am silent; it may, as you will see, be fairly surmised; but of actual proof there is none.

One thing, however, may, I think, be proved, and it is this—viz., that the natural meridian of the intellectual principle has not been witnessed, and that it frequently continues its progressive ascension, in spite of decay and decrepitude, up to the moment of corporeal annihilation. Let us not, therefore, confound insenescence with insenescibility.

This distinction is one of great importance; the latter term being the expression of a dogma, alike theoretical and paradoxical, and of which I have no need at present, while the word insenescence stands the representative of a general fact of historic origin, or of an *experimental* dogma, of which I propose to avail myself in considering several important points of human physiology.

Without dipping deeply into the science, but choosing rather some elevated point from which its principal divisions may be more readily appreciated, it is scarcely possible not to recognise six points upon which this insenescence of the intellectual principle serves to throw a palpable illumination. These are—1st. Legal medicine and medical ethics; 2ndly. The analysis of the human dynanism; 3rdly. The human synthesis; 4thly. The existing distinction between the nature of man and animals; 5thly. The appreciation of certain experiments made upon living animals with a view of elucidating the physiology of man; and, 6thly. The science of rational psychology, at present a subject ardently revived. A few words will serve to explain these different relationships.

1st. *Legal Medicine.*—Should it ever happen that legislators forget the reality of the insenescence of the intellectual principle, legal medicine would be there to remind them of it. You might say to me, that legislation has not ignored this fact, and that the acknowledged rights of old age are conformable to the above proposition, the legal presidentship in a deliberative assembly devolving upon the most ancient of its members. This is true, and we must confess that here common sense has triumphed over the theories of sages and philosophers.

But who will say that this victory over materialism is to be a permanent one, should its votaries unite *en masse*, and resolve to be consequent in the enunciation of their doctrines? Hence it should be the object of legal medicine to frame and elevate into a natural prerogative that which exists in reality; lest the dispensations of the law should ever range themselves side by side with the philosophy of the 18th century.

Let us, then, suppose that a popular belief continues tacitly to oppose the exertions of a sophistical science, and that the public feel persuaded that all things being equal, those intellects which have had the longest period of time for the exercise of their mental perceptions, deserve a preference in the consideration of matters requiring mature deliberation. Is this a reason for leaving unsolved the real motive of such conduct? No! It is of the utmost importance to reduce it to the standard of a mathematical formula, in order that sectarians may not deceive themselves with the notion, that so prudent a custom is, after all, merely an old prejudice, opposed to the dictates of reason and progress.

But, in addition to legal medicine, there exists a moral medicine, or code of medical ethics. We possess an inward tribunal, the distributive justice of which requires as much assistance from the science of medicine, as does that of our external tribunals. And a strict examination of our principle of intelligence is far more competent than public laws or public opinion to grant or deny certain dispensations. Whatever legal exemptions may be claimed in favour of age and infirmities, a conscientious individual, when he feels that his intellectual powers may escape the influence of years and of decay, will probe his own mind in order to appreciate truly both his duties and the powers he still possesses for their performance. In this way a knowledge of the fact we are now considering contributes to render us not only irreproachable, but at the same time exempt from all remorse.

The treatises of Cicero, St. Evremont, and Meister, are indeed a means of consolation, adapted for those who are advanced in years, but they are at the same time little short of manuals for the voluptuary, in which endeavours are made to attract his attention towards some pleasant notions, offered as compensation for the losses he may have experienced. And I fear that such epicurean morality can only tend towards the false idea, that age constitutes a dispensation from mental exercise. Sensuality may, in this way, exaggerate the rights of age at the expense of its moral obligations. It is necessary, therefore, that along with the mitigations required by the increasing debility of the vital powers, should be inscribed rules of conscience, deduced from the antagonistic and progressive career of the intellectual principle. As long as this principle is incapable of alleging a true declension, it must not be permitted to quit its post.

Man, in his civil capacity, is a citizen of two republics, of which the one is material—the other intellectual. The elder no longer pertains to the first of these, when his organic principle refuses to perform its former wonted services as in days past; he becomes nought but a parasite, and may reasonably dread the fate of the drones when the hive of bees no longer requires their presence. In the intellectual republic, however, he still preserves his rank and rights, and may, if he choose, do so up the latest term of his existence. The truth of the insenescence of the intellectual principle becomes thus an appeal, not only to his self-esteem, but likewise to that innate sense of duty which regulates an honest man, and whispers in his ear, that no sentiment capable of proving useful to humanity at large, deserves to be buried in the tomb.

2nd. *Analysis of the Human Dynamism.*—The insenescence of the principle of intelligence once established, we may proceed to utilise it in an examination of the nature of this principle, as compared with that of the vital force in man. You are aware that this latter power acts, presides, and suffers, unconscious of itself; while the very reverse is the case with the principle of intelligence. It is already a difficult matter to consider, as of an identical nature, two powers—the one of which is endowed with a consciousness, of which the other is devoid. But if, in addition, the latter is without exemption, prone to decay, while the former continues its onward progression for an indefinite period, and enjoys a real insenescence, shall we, I repeat, then say that these two great principles are alike? . . . . What becomes of the principles of metaphysics, or of the rudiments of philosophy, if phenomena so different, so distinct, allowed of our seeking their respective origin in a single hidden cause.

The history of man, then, is derived from a science itself of duplicate essence; the one division being of a physical nature, namely, anatomy; the other of a metaphysical nature, again subdivided into—first, psychology; and secondly, human biology. These three very distinct sections of physiology are all of equal importance, and alike deserving of conscientious careful investigation.

3rd. *The Human Synthesis.*—Those who are convinced of the difference which really exists between the primary causes from whence all anthropologic phenomena are derived, will allow, that since man is composed of three elementary principles, none of which can be considered as proceeding from the other, but are united in one system by virtue of a superior agency . . . . we should carefully inquire into the laws which regulate this association. These laws, not to be compared, indeed, with those of nature in general, are the subject of a special department in human physiology. They can only be known and reduced to anything like precision, by acquiring exact notions respecting the anatomy, psychology, and biology of man, and consequently, after having accurately distinguished the two elements of the human dynamism. The code of this association is what we denominate, anthropopœia; a science essentially medical, intimately connected with moral principles, and having no equivalent in comparative physiology.

A distinct appreciation of the three elements just mentioned, is by no means, however, the sole condition indispensable to a formal establishment of the laws which regulate their co-ordination; it is, in addition, necessary that we should be agreed upon the fundamental doctrines of philosophy, upon its axioms, and upon the technicalities which express them. For such a mutual understanding, it is necessaay to appreciate the legitimate signification of the word ontology. Now it was no more lawful for Brouissais to consider ontology (another term for general metaphysics) as the poetic creation of an imaginary entity, than it was for Prado to take the words metaphor and metonymy for terms in chemistry. Moreover, we must become familiarized with the rules of the "Novum Organum," which is but the manual of the art of philosophizing in the interpretation of nature. Experimental causes are not to be regarded in the light of hypotheses, inasmuch as the use of these abstract expressions has had for its object the exclusion of all concrete supposition. In distinguishing natural causes, it is of consequence to bear in mind the two heads to which they may be referred, the physical and the metaphysical. Lastly, in a specific determination of experimental causes, the reasoning should be as impartial as should be the heart in the verdict of a jury.

Without a due observance of these precepts, I see no possibility of arriving at an exact analysis of man; and without such an analysis, how can we hope to arrive at any rational mental synthesis. Hence it is that anthropopœism is not even dreamt of in those schools of physiology where the Hippocratic analysis is ignored and repulsed. Cabanis, who would appear to have attempted it, failed to achieve the desired end. Among many requisite conditions in which he was wanting, we may particularly remark the one I have just mentioned. Blinded by the spirit of materialism, he never possessed that independence of reasoning so necessary in applying the method of Bacon to the discrimination of experimental causes. The title of his work, "Du Rapport du

Physique et du Moral," is in itself a physiological solecism, since the moral bears no relation to the physical, properly speaking, but only to the vital, which alone influences the latter. Neither Bichat nor Broussais have been more attentive in their analysis than Cabanis. The science of medicine truly requires some other synthesis, under penalty of losing all philosophic bond of union.

4th. *Parallel of the Human Dynamism with that of Animals.*—I have already, on another occasion, instituted certain comparisons, the result of which has served to prove that the two dynamisms were not of an identical nature. If then the insenescence of the principle of intelligence be incontestable, you must no longer expect to see any greater resemblance between man and the brute.

If the brute be not endowed with a principle capable of resisting the decay of its vital force, I have a right to suppose that its dynamism is not the same as that of man; either it is simple in composition, or it is a combination of two elements, and if so, that which would be equivalent to our principle of intelligence must be essentially different. Already do we perceive the contrast between the delicate susceptibility of the human brain, of which trifling lesions are sufficient to threaten existence, . . . . and the tolerance of this organ in many animals; a tolerance, indeed, which allows it to be mutilated, and even partially destroyed, without much alteration of function: equally evident becomes the contrast which exists between so many animals which from their birth are enabled to supply their own wants without instruction, and man, who cannot live after birth but by the assistance of his fellow-creatures, or by the aid of a lengthened apprenticeship. Such contrasts, I repeat, must have often struck unprejudiced minds, as showing that the experimentalists were comparing things which did not admit of comparison, and that the cerebral physiology of animals could have but little in common with that of man. But the insenescence of the intellectual principle in the latter, as opposed to the senescence of the vital principle, and on the other hand, the complete degradation of the entire animal, should suffice, I think, to open all eyes, and to convince physicians that an amalgamation of human and animal physiology is, after all, a bastard production, of which no application can be made, whether in medicine psychology, or ethics.

5th. *Application of certain vivisections undertaken with a view of determining the functions of the Brain.*—One of the most serious results attending the establishment of the insenescence of the principle of intelligence is, that it destroys the value of those experiments which have been made on the brain and nerves of living animals, with the view of solving the theory of the intellectual faculties in man. I feel satisfied that the anticipation of such a result may prove the most active cause of opposition to my doctrines. What becomes of so many vivisections, so ingeniously imagined, so carefully and laboriously executed? And yet, what reliance can I place upon phenomena observed in beings so different from myself? You see that, to apply the results of vivisection to the elucidation of human physiology, it becomes necessary to prove that animals possess a principle of intelligence analogous to our own—a principle of intelligence that can resist the influence of years, and remain vigorous and unscathed while the body withers and the vital powers are becoming extinct. You must prove to me that in animals there exists some intellectual compensation as a set-off to the destroying influence of age upon their bodies; and unless you can do that, I must continue to deny the identity of their nature and mine.

Now, in spite of the difference which really does exist, we are perpetually told, that these vivisections are of the greatest use in the advancement of medical science.

I have, on another occasion, entered my protest against that doctrine which admits of a compromise between the science of man and comparative physiology. I have long since been scandalized at perceiving that the designation and privileges of the human being had become the common property of certain species, whose only claim to such consisted in their possessing, along with him, vertebræ, or some other anatomical peculiarity. . . . And this sentiment would indeed be heightened, if I found that our brethren allowed the laws of human psychology to be framed upon the result of experiments made upon the lobes, corpora striata, or tubercula quadragemina of rabbits, turkeys, fowls, &c. Believe me, the hierarchy of science is never overthrown or deranged with impunity. It has been determined beforehand by the dictates of common sense, and any infractions of its laws are sooner or later avenged.

6th. *Rational Psychology.*—Physiology, medically speaking, comprehends only that division of psychology denominated empiric psychology, or in other words, the science of the functions of the intellectual principle, without regard to the nature of such principle. But an inquiry, having for its object the solution of this problem, which is of little interest as regards our own peculiar department of science, becomes nevertheless of great importance in a moral as well as in a political and philosophical point of view.

The editors of the *Encyclopédie des Sciences Philosophiques,* professing to establish truths by the unaided powers of reason, without the intervention of faith; teach without reserve, that "the mind is a free and responsible agent, an existence entirely distinct from any other; self-containing, self-producing, self-governing, and bearing along with the stamp of its own origin the warrant of its own immortality."

I cannot imagine that these judges should have pronounced such a verdict without consulting us, who, as physicians, form a sort of public tribunal in relation to the science of man. They would rather wish to know our opinion of this matter in an experimental light. Now, is it not evident how the insenescence of the human intellectual principle comes to the assistance of their proposition? This insenescence, is it not a presage of insenescibility, and is not insenescibility a step on the road to indefectibility, and, as a consequence, to immortality?

The sect of the Organiciens, representing the philosophy of the eighteenth century, addresses itself to us in the same terms as did Augustus (after the taking of a city) to the inhabitants who craved his clemency—"Moriendum est," (death is inevitable.) But does nature, or to speak more definitely, the analysis of facts, speak as pitilessly as these philosophers? Is the literal interpretation of the sentence susceptible of no modification? May I not expect some slight commutation? Fortified by a conviction of the existing distinction between the two principles of my own dynamism, may I not console myself with the reflection that all is not lost, and that it is not unwarrantable to exclaim in a literal and experimental sense, as did the poet in a metaphysical one—"Non omnis moriar"?

From what I have said, gentlemen, you must, I think, feel persuaded that my proposition relative to the insenescence of the human principle of intelligence is not a frivolous paradox, incapable of application. Whatever contrary opinions you may happen to hear upon the subject, do not believe that it is merely a phantom of the imagination, or that, on the other hand, it is but an apology on my part, for an order of which I have long been a professed member, and which I now seek, from selfish motives, to elevate in public estimation.

Believe me, I am actuated by far higher motives. Once for all, be persuaded that it is my wish to teach nothing but fundamental truths. I am as economical of your time as I am of my own, and I fear not to remind you of the declaration of Bossuet, in which I see the expression of my own duty—the approval of my own sentiments. Though my body be feeble, the intellects of those around me still require direction. The good they may anticipate is indeed unworthy to be compared with that which the illustrious prelate was enabled to dispense. But though temporal and of an inferior order, it possesses a certain dignity, since it interests humanity at large. The sublimity of the one is no reason for depreciating the value of the other. If the former has been, metaphorically speaking, denominated the word of life, we cannot be reproached with applying a similar term to the latter, in an acceptation approximating more to its true and literal meaning.

It is, then, in the hope of disseminating these (I trust) beneficent doctrines, that each succeeding year finds me increasingly animated, in proportion as the chilling influence of age deadens my corporeal perceptions. Each day do I desire, more and more, to protect you from the ignorance and errors which threaten you, without considering that present and future inspectors, more able than I, will doubtless preserve you from them. You may carry home my words to your fathers, who have been heretofore my friends and auditors. I feel myself in the condition of one who, at the close of his earthly career, sees his rapidly approaching departure, and exaggerates the approaching misfortune of his young children. His grief arises not from a presumptuous estimation of his own capacity, as compared with that of those who may be their future guardians; but from an all-powerful conviction, that to none but himself can the happiness of his little ones ever be so dear.

Seek not, therefore, my beloved children and pupils, to destroy or dissipate an illusion which is, indeed, little short of providential; inasmuch as I owe to it a courage truly paternal, and expect on your parts a sentiment of gratitude susceptible even of imitating filial love.*

---

### DESCRIPTION OF A NEW BED AND BEDSTEAD FOR THE USE OF INSANE AND OTHER PATIENTS.

* Lecture II. will appear in our next number.

